# Simulation of the Effect of Artificial Water Transfer on Carbon Stock of* Phragmites australis* in the Baiyangdian Wetland, China

**DOI:** 10.1155/2017/7905710

**Published:** 2017-02-28

**Authors:** Xinyong Chen, Fengyi Wang, Jianjian Lu, Hongbo Li, Jing Zhu, Xiaotong Lv

**Affiliations:** ^1^Shanghai Key Laboratory for Urban Ecological Processes and Eco-Restoration, School of Ecological and Environmental Sciences, East China Normal University, Shanghai 200062, China; ^2^Hebei Provincial Academy of Environmental Sciences, Shijiazhuang City, Hebei 050037, China; ^3^State Key Laboratory of Estuarine and Coastal Research, East China Normal University, Shanghai 200062, China; ^4^Hebei Zhengrun Environmental Technology Co. Ltd., Shijiazhuang City, Hebei 050000, China

## Abstract

How to explain the effect of seasonal water transfer on the carbon stocks of Baiyangdian wetland is studied. The ecological model of the relationship between the carbon stocks and water depth fluctuation of the reed was established by using STELLA software. For the first time the Michaelis-Menten equation (1) introduced the relation function between the water depth and reed environmental carrying capacity, (2) introduced the concept of suitable growth water depth, and (3) simulated the variation rules of water and reed carbon stocks of artificial adjustment. The model could be used to carry out the research on the optimization design of the ecological service function of the damaged wetland.

## 1. Introduction

Water has an important role in the growth of wetland plants [1–4]. Changes in wetland water content influence surface vegetation type and individual form, which in turn affect both the demand for and the utilization efficiency of water. Because water is involved in processes such as plant photosynthesis and respiration, it can affect directly or indirectly the fixation and release of CO_2_ by vegetation [5–7]. Wetland plant biomass reflects the fixed shape, size, and productivity of ecosystem carbon [5]. Thus, changes in artificial or ecological water, or other human activities that alter the hydrological situation of wetlands, can influence wetland photosynthetic processes and directly or indirectly affect vegetation carbon reserves.

Artificial water transfer has become one of the most direct and effective approaches for the management of water quantity in wetlands, especially in temperate regions and in areas with scant water resources, for example, the Baiyangdian wetland, Zhalong wetland, and Lake Wuliangsuhai in China. Each year, water in these areas is regulated artificially to ensure the appropriate water depth to maintain wetland ecological function [8]. Previous research of vegetation growth in wetland ecological systems has elucidated many aspects of the carbon cycle [9–14]. However, most studies have considered natural wetlands rather than those affected by artificial water transfer. Therefore, it is necessary to investigate the effect of the artificial regulation of water depth on wetland plant growth and to consider the relationship between plant carbon stock and water depth fluctuation, to establish the consequences on the carbon cycle and carbon flux of wetlands.

Usually, the process of photosynthesis is expressed using the Michaelis-Menten equation [2, 4, 13, 14]. Based on previous research, this study introduced into the Michaelis-Menten equation the relation between water depth and the environmental carrying capacity of reeds, to elucidate the effect of environmental capacity on the carbon stock of reeds (biomass carbon content) in terms of water depth fluctuation.

In this paper, the most important hydrological conditions are studied, which are also the most easily controlled and influenced by human factors, which are one of the most effective means to restore the damaged wetland ecosystem services. The Baiyangdian* Phragmites australis* (reed) wetland under the influence of artificial water transfer is studied, and the ecological model of the relationship between the carbon stocks and water depth fluctuation of the reed was established by using STELLA software. We try to understand how to explain the effect of seasonal water transfer on the carbon stocks of Baiyangdian wetland and how to control the water transfer time and water transfer quantity (water depth) in Baiyangdian.

## 2. Study Area and Method

### 2.1. Study Area

**Figure 1:**
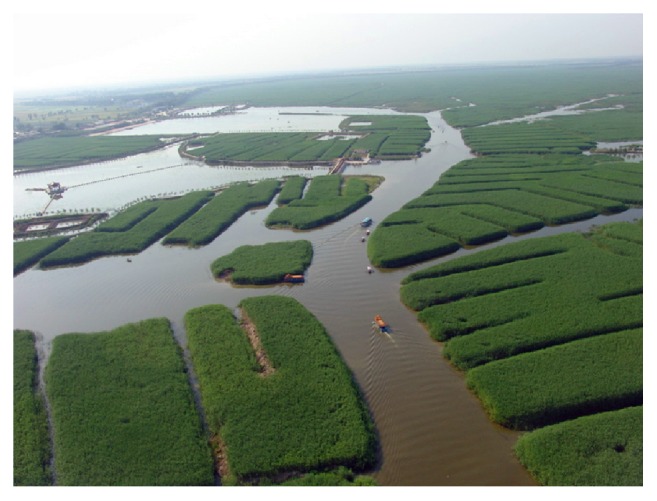
Typical landscape pattern of Baiyangdian wetland, phragmites platform.

Baiyangdian wetland is located in the middle of Hebei Province in China. It is the largest freshwater lake wetland of the North China Plain (38°43′–39°02′N, 115°38′–116°07′E), covering an area of 366 km^2^ (Dagu water level 10.5 m [15], which is zero in 1902 in Tanggu of Tianjin setting up a tide gauge as the basis). This area has a temperate continental monsoon climate that is cold and dry in winter but hot and wet in summer. The wetland area has more than 3700 ditches between connected platforms of* Phragmites australis* and 143 lakes (Figure 1). Its elevation is about 7.5–8.5 m and the average water depth is 1.0–2.0 m. Apart from a small number of poplar and peach trees planted on the edge of the Baiyangdian wetland, the majority of vegetation of this wetland ecosystem is* P. australis*, and this comprises the main resource for the local community.

Since the 1990s, due to the impact of environmental changes and human factors, the water depth in the Baiyangdian wetland has exhibited annual and seasonal fluctuations. The monthly fluctuation in the range of water depth in drought and wet years is 0.93–1.04 m and 1.35–1.56 m, respectively [16]. When the groundwater depth is <6.5 m (Dagu), it is called semidry and when it is <5.5 m (Dagu), it is called dry [17] (where level 6.5 m in Baiyangdian wetland means the water depth is about zero meters). In order to maintain the minimum ecological water level (7.3 m) [17] necessary to preserve ecological services in the Baiyangdian wetland, the local government implements an ecological water replenishment project. According to statistics (2001–2011), average annual replenishment with 80 million cubic meter of water [8] was required to maintain the Baiyangdian wetland water level at 6.6–8.6 m [18] (Dagu 8.6 m). To a certain extent, artificial water transfer modifies the hydrological situation of the Baiyangdian wetland, and it affects both the structure of the wetland and the growth status of the reeds. Therefore, it is necessary to determine the optimum time to implement artificial water transfer to maintain the water level within the prescribed design parameters and to have maximum effect on the carbon stock capacity of the reeds, in order to restore and maintain wetland ecological service function.

### 2.2. Data Sources

Carbon stock validation data was assessed in reeds sampled from June to 10th month of 2009 from near Yuanyang Island in the Baiyangdian wetland that was published by Li et al. [19]. The initial values of the aboveground and underground carbon stock of the reeds and the carbon stock of the litter were obtained by analogy [15]. The relationship between the aboveground carbon stock and the average water depth in the growing season was verified in 2010 by Zhao [18]. (In 2009 and 2010, the growth of the Baiyangdian wetland reeds reflected water level.)

This study collated ground vegetation carbon stock data from the Baiyangdian wetland (Shihoudian) [20], Zhalong wetland [21], and Sanjiang source* angustifolia* meadow wetland [22] to test the model.

Environmental temperature, solar radiation, and other parameters were obtained from the local meteorological bureau of Anxin County [15]. Details of the hydrological conditions and water transfer data were obtained from the Baoding Municipal Water Affairs Bureau.

## 3. Ecological Model Description

Plants absorb atmospheric carbon dioxide (CO_2_) via photosynthesis, which is then transformed and fixed in the plant. During life, the plant body transfers carbon from the rhizome of the root to the ground, and plant respiration releases carbon into the atmosphere [14, 23]. The decay of plant litter and of the root and stem after the death of the plant releases carbon into the soil [24]. In this study, based on the carbon cycle process, the STELLA software (version 9.1.3) was used both to build the relationship model between the underground carbon stock and water level fluctuation and to simulate the changes of carbon stock. The model was established with two state variables: aboveground tissue carbon stock (PaC) and underground carbon stock (PbC) (unit: gC/m^2^). The unit of the modeled process was gC/m/d, and the period of simulation was April 1 to October 31.

**Figure 2:**
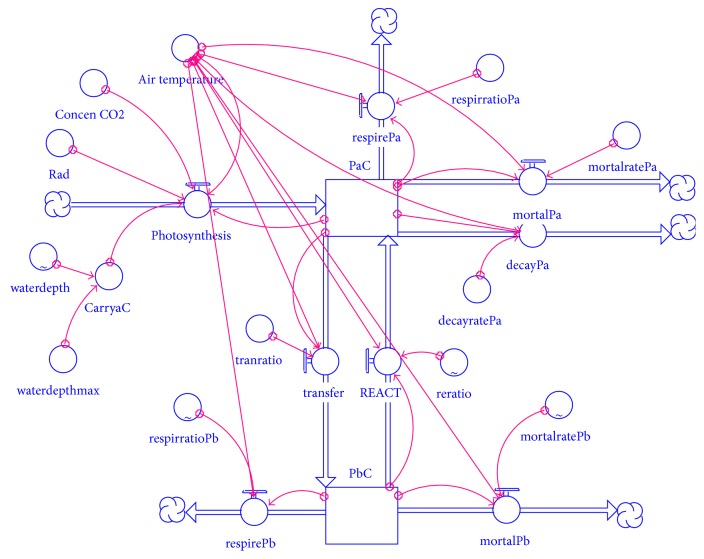
Conceptual diagram of STELLA model.

The forcing function of the model was CO_2_ concentration in ambient air, ambient temperature, solar radiation, and water depth. Abbreviations summarizes the symbols used in the model, their meanings, and their units. All processes in the model were affected and controlled by temperature. The temperature in this study was expressed using the Arrhenius equation. Temperature, water depth, rate of CO_2_ release, rate of plant litter organization on the ground, underground death rate, reed carbon reserves from the earth to the underground transport rate, and reed root on tissue like reactivation rate parameters are all time-dependent dynamic parameters on which the STELLA model was formulated.  Figure 2 shows the conceptual schematic of the STELLA model (see Abbreviations for explanation of the symbols).

### 3.1. Ecological Model Process and Equations

#### 3.1.1. Photosynthesis Process

The earth has only green plants (and photosynthetic bacteria) through photosynthesis and light interception energy directly from the sun, and they will use inorganic material (carbon dioxide) reduction of organic matter, as their own food. Reed leaf photosynthesis, that is, the use of solar radiation for CO_2_ assimilation of carbohydrates, is the most important source of energy in the ecosystem and the most important process in the model, which is expressed using the Michaelis-Menten equation [13, 25]. The Michaelis-Menten equation can change effects of solar radiation and air CO_2_ concentration on photosynthetic process; the rate of plant photosynthesis changes with atmospheric CO_2_ concentration and thus a constant semisaturated concentration of 300 ppm was introduced here [25], and it was considered that the vegetation would capture about 6% of the solar radiation available to the plant biomass [26].

The photosynthesis process of reeds was expressed by logistic growth, and the concept of environmental carrying capacity (CarryaC) was introduced, where we use the logistic equation to limit growth. That is, the aboveground carbon stocks of the reed are not of exponential growth but rise to the maximum smoothly. Because the growth of reeds is restricted by space and nutrient resources, the ultimate reed growth reaching the maximum capacity of the environment will slow down; this is the ecological significance of logistic equation. According to relevant research [4, 14], the process should be applied to the first-order equation and the influence of factors (1−PaC/CarryaC) on the organization of carbon stock/environmental carrying capacity. When the aboveground carbon stock of the reed reaches the limit of the environmental carrying capacity, the reed will stop growing. The model assumed that the maximum value of organic carbon in the reed was 2450 gCm^−2^ [18, 27, 28].

In this study, the relationship between water depth and environmental carrying capacity was introduced for the first time, that is, CarryaC is a function of water depth. The suitable growth water depth was introduced for the water depth of the wetland, that is, the minimum ecological water depth. Monitoring data of average water depth during the growth season of the reeds in the Baiyangdian wetland [18], other related research (showing the minimum ecological water level of 7.3 m, corresponding to the water depth of 0.8 m) [16, 17], and the special characteristics of the Baiyangdian reeds indicate that the water depth of 0.8 m is suitable for reed growth.

#### 3.1.2. Reactivation of the Underground Root of the Reed by the Aboveground Tissue

The reactivation process (REACT) of the aboveground tissue on the underground tissue of the reed rhizome begins during the initial stage of the growth season. It is a function of the carbon stock, reactivation rate, time, and temperature of the underground structure of the reed.

#### 3.1.3. Transmission Process of Aboveground Organic Matter of the Reed to Underground Tissue

As plant leaves begin to grow, the transmission process (transfer) of organic matter produced by photosynthesis to underground tissue occurs continuously throughout the growing season. After the cessation of photosynthesis, the energy of the leaf and stem of the reed [14] is redistributed underground. The process of energy transfer from aboveground to underground is a function of the carbon stock, transfer rate, time, and temperature of the plant, which is expressed in a first-order reaction equation in the model.

#### 3.1.4. Death Process of the Aboveground Tissue of the Reed, Decay of Litter, and Death of Underground Tissue of the Reed

The processes of mortalPa, decayPa, and mortalPb are all functions of the carbon stock, death rate, apoptosis rate, time, and temperature, which are expressed by first-order reaction equations.

#### 3.1.5. Respiration Process of Aboveground and Underground Tissue of the Reed

The processes respirPb and respirPa are determined by temperature, respiration rate, and carbon stock, which are expressed in the model with firs-order reaction equations.

The basic equations of the ecological model are shown as follows.

Basic equation
  *d*PaC/*dt* = PaC(*t* − *dt*)+(Photosynthesis + react − transfer − respirPa − decayPa − mortalPa)*∗dt*
  *d*PbC/*dt* = PbC(*t* − *dt*) + (transfer − react − respirPb − mortalPb)*∗dt*
Photosynthesis process
  Photosynthesis = Concen_CO_2_/(Concen_CO_2_ + 300)*∗*(Rad/(Rad +  6))*∗θ*^∧^(Air_temperature −20)*∗*PaC*∗*(1 − PaC/CarryaC)
CarryaC and water depth relation function
  If water depth = 0.8 then CarryaC = 2450 else CarryaC = 2450*∗*(waterdepthmax − ABS((waterdepth − 0.8)/2))/waterdepthmax
Retransmission process
  Transfer = tranratio*∗*PaC*∗θ*^∧^(Air_temperature − 20)
Reactivation process:
  REACT = reratio*∗*PbC*∗θ*^∧^(Air_temperature − 20)
Respiration process
  respirPa = mrespiratioPa*∗*PaC*∗θ*^∧^(Air_temperature − 20)
  respirPb = mrespiratioPb*∗*PbC*∗θ*^∧^(Air_temperature − 20)
Litter and death process
  mortalPa = mortalatePa*∗*PaC*∗θ*^∧^(Air_temperature − 20)
  decayPa = mortalratePa*∗*PaC*∗θ*^∧^(Air_temperature − 20)
  mortalPb = mortalatePb*∗*PbC*∗θ*^∧^(Air_temperature − 20)

## 4. Results

### 4.1. Model Parameters

Research on the dynamic changes of aboveground and underground reed carbon stock provides a large amount of data for the calibration of the ecological model. Other parameters used in the model calibration were derived from literature [14, 24, 29–32]. The model was calibrated by repeated testing until the parameters demonstrated good agreement with observed values.  Table 1 summarizes the parameter values of the model after calibration.

### 4.2. Model Verification

**Figure 3:**
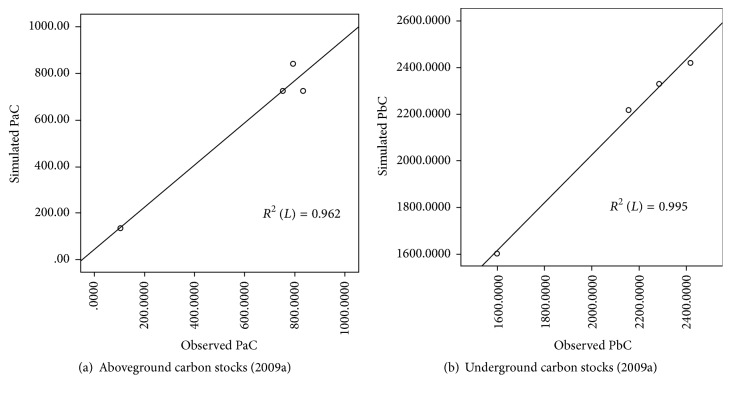
Comparison of observed and simulated carbon stock in Baiyangdian wetland in 2009: (a) aboveground plant tissue and (b) belowground plant tissue.

Based on the model calibration results, the observed and simulated values were compared and analyzed. Figures 3(a) and 3(b) show the results of the verification of the carbon stock in the aboveground and underground tissues of Baiyangdian reeds. The modeled value for 2009 is in good agreement with the observed value, reflecting the change of carbon stock of the reed during the growing season [20, 28]. The reeds grew steadily during the first three to four months of the growing season and then grew rapidly during months four and five, after which growth appeared to stabilize. The aboveground carbon stock reached its peak in August and underground carbon stock peaked in October.

**Figure 4:**
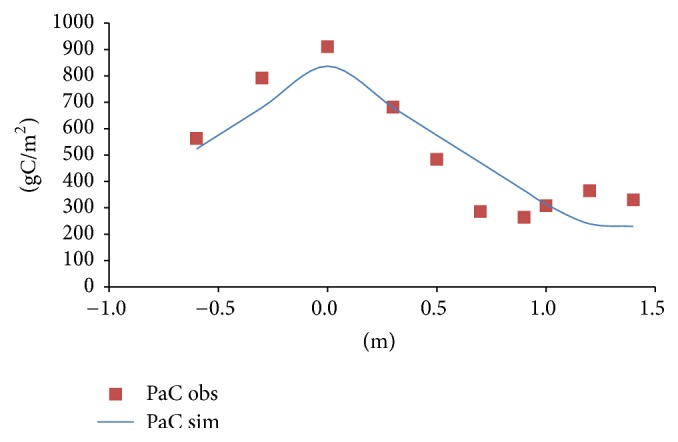
Comparison of the relationships of the simulated and observed carbon stock of aboveground plant tissue of reeds and water depth of the Baiyangdian wetland in 2009.

Figure 4 shows that the relationships of the simulated and observed aboveground carbon stock of reeds with water depth in the Baiyangdian wetland are in good agreement. Furthermore, it can be seen that an increase or decrease of depth relative to the suitable growth water depth of the reed caused a reduction of carbon stock. This could reflect the tissue carbon stock related to the growth of the reed platform is associated with seasonal changes of the average water depth. The 0 m relative water depth in the Baiyangdian wetland is defined as the suitable growth water depth of the reed [18] (corresponding water depth of 0.8 m), which is consistent with the largest carbon stock of the reeds.

### 4.3. Model Validation

**Figure 5:**
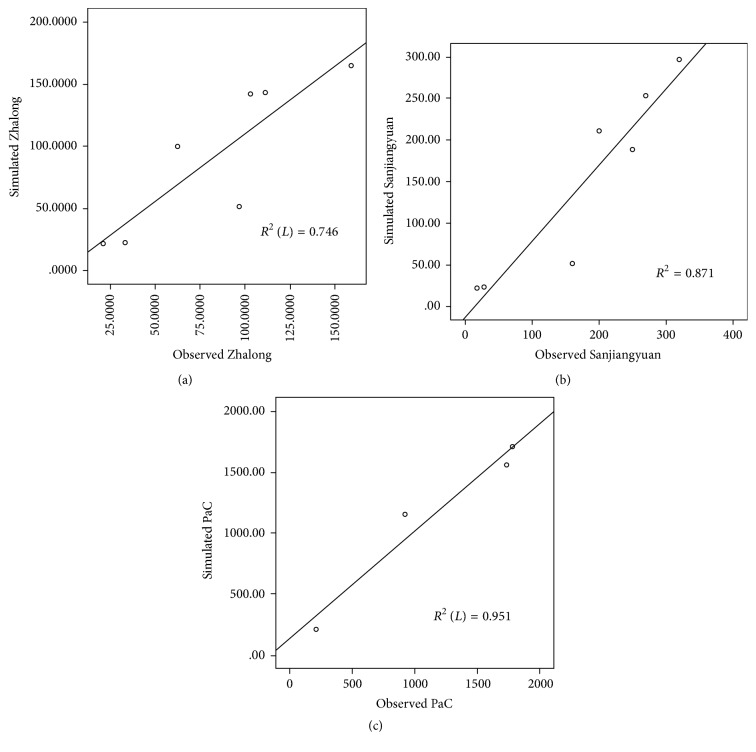
Comparison of observed and simulated aboveground carbon stock: (a) Zhalong wetland in 2010, (b) Sanjiang Plain wetland in 2012, and (c) Baiyangdian wetland in 2012.

The model was applied in the Zhalong wetland (Figure 5(a), 2010), Sanjiang Plain wetland (Figure 5(b), 2012), and Baiyangdian wetland (Figure 5(c), 2012). Analysis established that with suitable adjustment of the temperature, respiration, and release rate parameters of the model, the change trends of the simulated and observed values aboveground carbon stock were consistent. Thus, this model could be applied in equivalent calculations for similar wetlands.

### 4.4. Simulation Prediction

Based on the model validation and verification, the water depth conditions of different seasons (within the range of 1.0 m) were set, and the conditions of artificial water transfer in the Baiyangdian wetland were simulated. The change trend of reed carbon stock was predicted for the entire growing season under different water depths (i.e., 0.1, 0.3, 0.5, and 0.8 m) and in different seasons (spring, summer, fall, winter harvest, and treatment) with different water depths (0.1, 0.3, 0.5, and 0.8 m).

#### 4.4.1. Entire Growing Season

**Figure 6:**
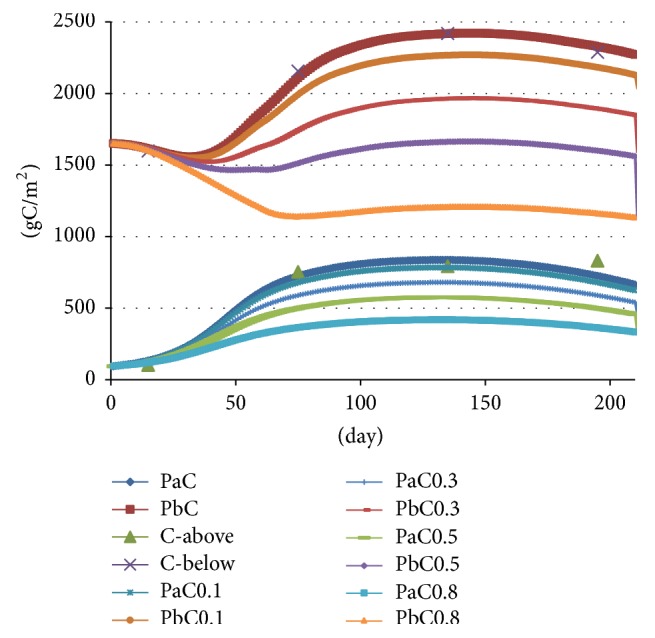
Change trend of carbon stock in aboveground/underground tissue of reeds during the entire growing season.

Under conditions of suitable growth water depth, fluctuation of water depth caused by water transfer will not be conducive to reed growth and there will be a decrease in carbon stock (Figure 6). For water depth change of ±0.1 m, the corresponding changes of carbon stock of aboveground and underground are from −1.8% to −9.4% and from 0.2% to −9.8%, respectively. For water depth change of ±0.3 m, the corresponding changes of carbon stock of aboveground and underground tissue are from −1.8% to −29.5% and from 0.2% to −29.8%, respectively. For water depth change of ±0.5 m, the corresponding changes of carbon stock of aboveground and underground tissue are from −1.8% to −49.6% and from 0.1% to −49.9%, respectively. For water depth change of ±0.8 m, the corresponding changes of carbon stock of aboveground and underground tissue are from −1.8% to −50.0% and from 0.0% to −50.0%.

The simulation results indicate that artificial regulation of water has considerable effect on carbon content during the growth season of the reeds. Therefore, water depth increases due to artificial water transfer which should be limited to within 0.5 m (preferably to within 0.3 m) in order to reduce the influence of water depth fluctuation on the carbon stock of the reeds.

#### 4.4.2. Different Seasons

**Figure 7:**
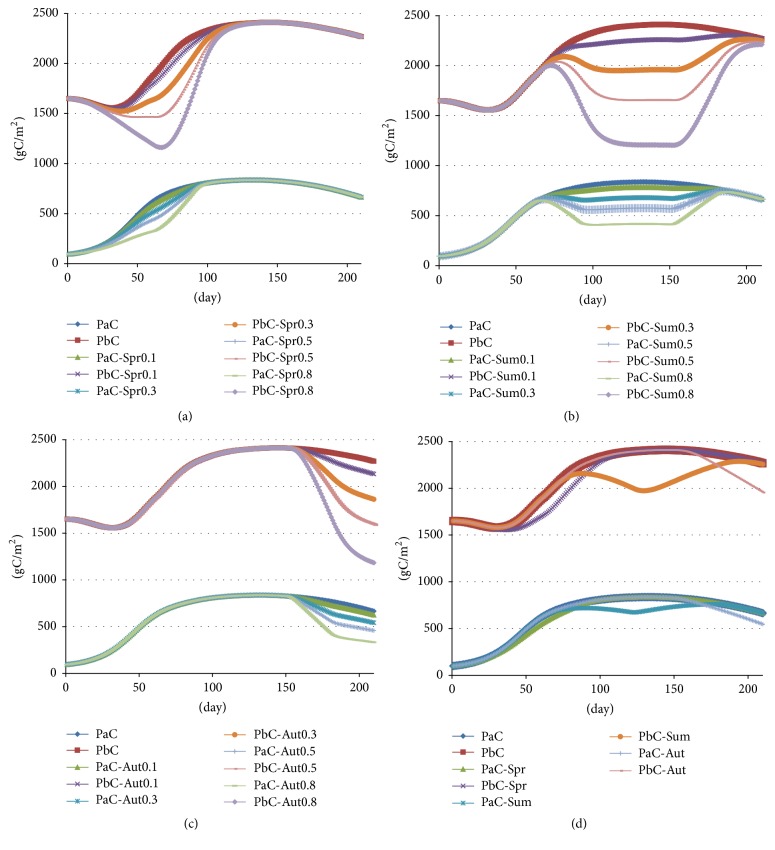
Change trend of carbon stock in aboveground/underground plant tissue: (a) spring, (b) summer, (c) fall, and (d) entire growing season.

Figure 7(a) shows that artificial water transfer in spring caused a reduction in the carbon stock of the reeds during the same period (the largest reduction in aboveground and underground carbon stock was from −5.9% to −48.3% and from −5.0% to −42.1%, resp.), consistent with other research results [1, 4, 35, 36]. However, toward the end of the flood season, or when the water depth reverted to the suitable growth water depth, the ability of the reeds to store carbon was gradually restored by summer. Obviously, depth increases due to artificial water transfer in spring which should be controlled to within 0.5 m (preferably to within 0.3 m).

Figure 7(b) shows that artificial water transfer in summer also caused a reduction of reed carbon stock (the largest reduction in aboveground and underground carbon stock was from −6.2% to −50.0% and from −6.2% to −49.9%, resp.). However, toward the end of the flood season, or when the water depth reverted to the suitable growth water depth, the ability of the reeds to store carbon was gradually restored by the beginning of fall. However, the degree of flooding dictates whether it can return to the value. Thus, depth increases due to artificial water transfer in summer which should be controlled to within 0.5 m (preferably to within 0.3 m).

Figure 7(c) shows that artificial water transfer in fall also caused a reduction in reed carbon stock until the end of season (the largest reduction in aboveground and underground carbon stock was from −6.2% to −50.0% and from −6.1% to −49.5%, resp.). However, subsequently, reed carbon stock was not restored. Again, the conclusion can be drawn that depth increases due to artificial water transfer in fall which should be controlled to within 0.5 m (preferably to within 0.3 m).

Based on the above simulation, Figure 7(d) illustrates the overall change trend of reed carbon stock in the growing season when the water depth fluctuated by 0.3 m. The results show that water depth fluctuations caused by artificial water transfer resulted in a decrease of carbon stock compared with the same period when water depth was regulated to the suitable growth water depth. Artificial water transfer in spring was relatively less; however, in the latter stage with the return of the water depth, the growth of the reeds might be gradually restored. Therefore, artificial water transfer is important for maintaining the suitable growth water depth in the Baiyangdian wetland, which enables reed growth and the increase of reed carbon stock.

## 5. Discussion

1. Usually, the process of photosynthesis is expressed using the Michaelis-Menten equation [2, 3, 12–14]. Based on previous research, this study introduced into the Michaelis-Menten equation a relation between the environmental carrying capacity of reeds and water depth, as well as the concept of suitable growth water depth. A model of the relationship between the carbon stock capacity of reeds and water depth fluctuation was constructed, and simulations of the Baiyangdian wetland were performed. The results showed good agreement between the predicted and observed values, reflecting the variation of reed carbon stock with water depth. Thus, this model provides a new approach to the simulation of the growth of water plants in different water depths.
2. This study established that the influence of water depth fluctuation on the reed carbon stock is seasonal (spring, summer, and fall); however, a general decrease of the carbon stock was found. Furthermore, it was revealed that water flooding affects the photosynthesis process, which inhibits/delays reed growth [4, 33–35]. Similarly, in summer and fall, water diversion was found to cause a decrease in reed carbon stock, confirming the results of many other studies [21, 36, 37]. The results of this study showed that an appropriate volume of artificial water transfer is required to maintain the suitable growth water depth, which is conducive to reed growth and to the increase of carbon stock.
3. Based on the findings of this study, in order to maintain or increase the carbon stock of the Baiyangdian wetland reed, it is suggested that spring is the optimum time for artificial water transfer. Fall is not an appropriate season for water transfer, and if conducted in summer, it should be performed late in the season (i.e., the end of August). The range of water depth fluctuation due to artificial water regulation should be limited to within 0.3 m.
4. The growth and carbon cycles of wetland plants are extremely complex and they are influenced by many factors. In this study, the most important hydrological conditions were selected, which also constitute those factors most vulnerable to human control and influence. Thus, improved understanding of these factors is most effective for the restoration of damaged wetland ecosystem service function. This study focused on the artificial regulation of water, within the context of seasonal fluctuations of water depth and the carbon stock capacity of the Baiyangdian wetland, to draw the relevant conclusions; however, the effects of spatiotemporal scale and environmental and human factors should be considered in future research.

## 6. Conclusions

This study considered the influence of seasonal fluctuations of water depth and artificial water transfer on the carbon stock of the reeds in the Baiyangdian wetland. Based on this analysis, a relational model was established, which was used to simulate the effects of the artificial regulation of water on the growth of reed carbon stock. It is of considerable importance to estimate accurately the cycle and flux of carbon under the influence of artificial control. It was demonstrated that the model could be used in the design of the ecological service function of damaged wetlands and that it could be applied to other similar wetland environments.

There are three main conclusions to this research. (1) For the first time, a relational function between the environmental carrying capacity of reeds and water depth, as well as the concept of suitable growth water depth, has been introduced into the Michaelis-Menten equation. Thus, this provides a new approach to the simulation of the growth of water plants in different water depths. (2) The results showed that an appropriate volume of artificial water transfer is necessary to maintain the suitable growth water depth, enabling reed growth and the increase of carbon stock. (3) The optimum time for artificial water transfer in the Baiyangdian wetland is spring. The range of water depth fluctuation due to artificial water regulation should be limited to within 0.3 m.

## Figures and Tables

**Table 1:** Basic parameters used in the model.

Parameters	Range	Unit	Source
INIT Pac = 103	100*～*150	gCm^−2^	Libo 2012;
INIT Pbc = 1650	1500*～*2000	gCm^−2^	Libo 2012;
INIT Concen CO_2_ = 400	280*～*404.83	ppm per year	http://www.carbonify.com/carbon-dioxide-levels.htm
*θ* ^a^ = 1.05*～*1.09			Calibrate and Calibrate;
CarryaC = 2450	1000*～*5000	gCm^−2^	Libo 2012; Zhao 2012; Guo 2012; Zhang et al. 2014;
waterdepthmax = 1.8	0.0*～*2.0	m	Zhao et al. 2005; Cui et al. 2010; Zhao 2012;
Rad = 14.67	14.67	MJ m^−2^ per year	Collect;
decayratePa = 0.005	0.0*～*0.18	gg^−1^ per day	Calibrate;
mortalratePa = 0.001	0.0*～*0.15	gg^−1^ per day	Soetaert et al. 2004, Eid et al. 2012;
mortalratePb = 0.001	0.0*～*1.0	gg^−1^ per day	
transratio = 0.20	0.0*～*0.35	gg^−1^ per day	Zhang et al. 2014;
carbratio = 0.4	0.0*～*0.5	gg^−1^ per day	Calibrate;
mathratio = 0.006	0.0*～*0.3	gg^−1^ per day	Calibrate;
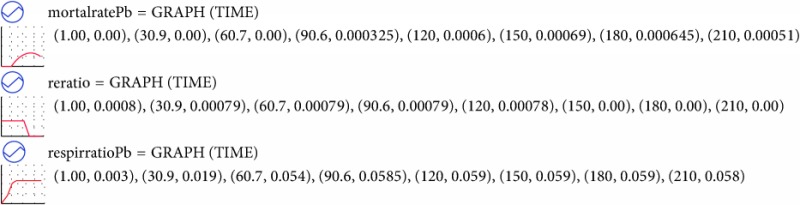			

^a^ *θ* is the Arrhenius constant, Soetaert et al. 2004, Zhang et al. 2014, and Jørgensen and Nielsen 2015; *θ* is 1.09 in photosynthesis and respirePb; *θ* is 1.07 in respirePa, transfer, and REACT; *θ* is 1.05 in mortalPa, mortalPb, decayPa, methane emission, and carbon emission.

## Abbreviations

### Summary of Symbols Used in the STELLA Model

PaC: Aboveground carbon stocks of the reed (gCm^−2^)
PbC: Underground carbon stocks of reed (gCm^−2^)
Photosynthesis: Photosynthesis (gCm^−2^ per day)
decayPa: Litter carbon stocks in aboveground tissues of the reed (gCm^−2^ per day)
mortalPa: Aboveground tissue death carbon stocks (gCm^−2^ per day)
mortalPb: Underground tissue death carbon stocks (gCm^−2^ per day)
respirePa: Aboveground tissue respiration of reed (gCm^−2^ per day)
respirePb: Underground tissue respiration (gCm^−2^ per day)
REACT: Remobilization (gCm^−2^ per day)
transfer: Translocation (gCm^−2^ per day)
Air temperature: Air temperature (°C)
Concen CO_2_: Air carbon dioxide concentration (ppm per year)
CarryaC: Maximum environmental carrying capacity of reed (gCm^−2^)
Rad: Solar radiation (MJ m^−2^ per year)
(Suitable) water depth: Suitable water depth/average depth (m)
waterdepthmax: Maximum water depth (m)
decayratePa: Tissue litter rate on the ground of reed (gg^−1^ per day)
mortalratePa: Death rate of the aboveground reed (gg^−1^ per day)
mortalratePb: Death rate of the underground reed (gg^−1^ per day)
respirratioPa: Respiration rate of the aboveground reed (gg^−1^ per day)
respirratioPb: Respiration rate of the underground reed (gg^−1^ per day)
reratio: Remobilization rate (gg^−1^ per day)
transratio: Translocation rate (gg^−1^ per day).
